# Heavy Metals Assessment and Health Risk to Consumers of Two Commercial Fish Species from Polyculture Fishponds in El-Sharkia and Kafr El-Sheikh, Egypt: Physiological and Biochemical Study

**DOI:** 10.1007/s12011-023-04007-1

**Published:** 2023-12-22

**Authors:** Mahmoud Mahrous M. Abbas, Salah M. EL-Sharkawy, Hassan R. Mohamed, Bassem E. Elaraby, Walaa M. Shaban, Metwally G. Metwally, Diaa M. G. Farrag

**Affiliations:** 1 Marine Biology Branch, Zoology Department, Science Faculty, Al-Azhar University, Cairo, Egypt; 2 Zoology Department, Science Faculty, Al-Azhar University, Cairo, Egypt; 3 Marine Products Processing Technology Department, Aquaculture and Marine Fisheries Faculty, Arish University, Arish, Egypt

**Keywords:** Heavy metals, Correlation, Bioaccumulation, Aquaculture, Pollution index, Immunological parameters, T-test

## Abstract

Metal pollution is a major environmental concern worldwide, especially in Egypt. The aquaculture industry uses widespread artificial feeds to stimulate fish production, leading to metal accumulation in the aquatic environment. Heavy metal concentrations (HMCs) in sediments, water, and tissues were studied to study the effect of pollution levels on heamatological, and biochemical, immunological aspects of farmed fish as well as on human health. Results declared that the HMC levels in the water and sediment were significantly different between El-Sharkia and Kafr El-Sheikh fishponds (T-test, *p* < 0.05). This was supported by the metal pollution index in the water and sediment, indicating that El-Sharkia fishponds (ES fishponds) were more contaminated than Kafr El-Sheikh fishponds (KES fishponds). Also, HMCs in fish tissues were significantly increased in fish cultivated in ES fishponds than in KES fishponds. Haematological, immunological, and biochemical alterations of Bolti (*Oreochromis niloticus*) and Topara (*Chelon ramada*) fish were significantly different within the different fish species as well as the different fishponds. From the human health perspective, the THQ-HMC and HI-HMC associated with the consumption of muscle suggest a safe non-carcinogenic risk to human health. In contrast, cadmium poses a cancer risk to children who consume the muscular tissue of Bolti fish from ES fishponds, which should be regarded as a warning sign based on data indices and a human health perspective. In order to minimise HMC pollution in the aquaculture sector, it is advisable to take possible assessments and carry out continuous monitoring considering international WHO/FAO assessments.

**Supplementary Information:**

The online version contains supplementary material available at 10.1007/s12011-023-04007-1.

## Introduction

Aquaculture production is increasing every year worldwide and in 2018, 179 million tons of aquaculture output were produced. It is believed that increasing fish production is the only possible option to close the gap between protein production and consumption [26, 41]. In Egypt, the majority of aquaculture farms use Bolti (*Oreochromis niloticus*) and Topara (*Chelon ramada*) as part of their polyculture practices [28, 30].

The HMCs are present in aquatic habitats in a variety of ways, including through untreated or insufficiently treated agricultural, domestic, and industrial effluent [80]. Metals are ingested by aquatic organisms in small amounts through water uptake and in larger amounts through biomagnification of prey,however, consumers can ingest metals through the food chain, which can have acute and long-term health effects [3]. Egyptian aquaculture farms serve as temporary reservoirs for wastewater Soltan et al. [61], with the aquatic habitat and its water quality being the primary determinants of fish health or disease. One of the main threats to aquatic organisms is pollution of the aquatic environment by inorganic and organic pollutants [57].

The assessment of the quality of the aquatic environment via monitoring the levels of HMC pollution by determining their levels in sediment, water, and tissues of fish [9, 34]. The fish HMCs are significantly influenced by several factors, and identifying fish species with elevated levels of metals is very important for consumer awareness and safety The level of contamination varies by fish species, source of contamination, trophic level, collection site, and type of feeding [74]. Physiological and biochemical indices in aquatic organisms are widely used as biomarkers to assess their health [23, 29]. Due to the risks to human health connected with the bioaccumulation of HMC through fish intake, the monitoring of HMC in fish and fish farm environments has become crucial. Consequently, it is important to determine the HMC in the muscles of cultivated fish to assess the potential risks of their intake for humans. Therefore, this study aims to (i) evaluate the quality status of the polyculture ponds (KES and ES fishponds) regarding ecotoxic HMC considering three interrelated aquatic compartments: water, sediments, and fish species )Botti, and Topara(, (ii) study the effect of pollution levels on heamatological, biochemical, and immunological, aspects of ponds fish and (iii) to evaluate the possible human health hazards associated with ingestion of muscle of ponds species by applying the non-carcinogenic and carcinogenic equations.

## Material and Methods

### Sampling Fishponds

**Fig. 1:**
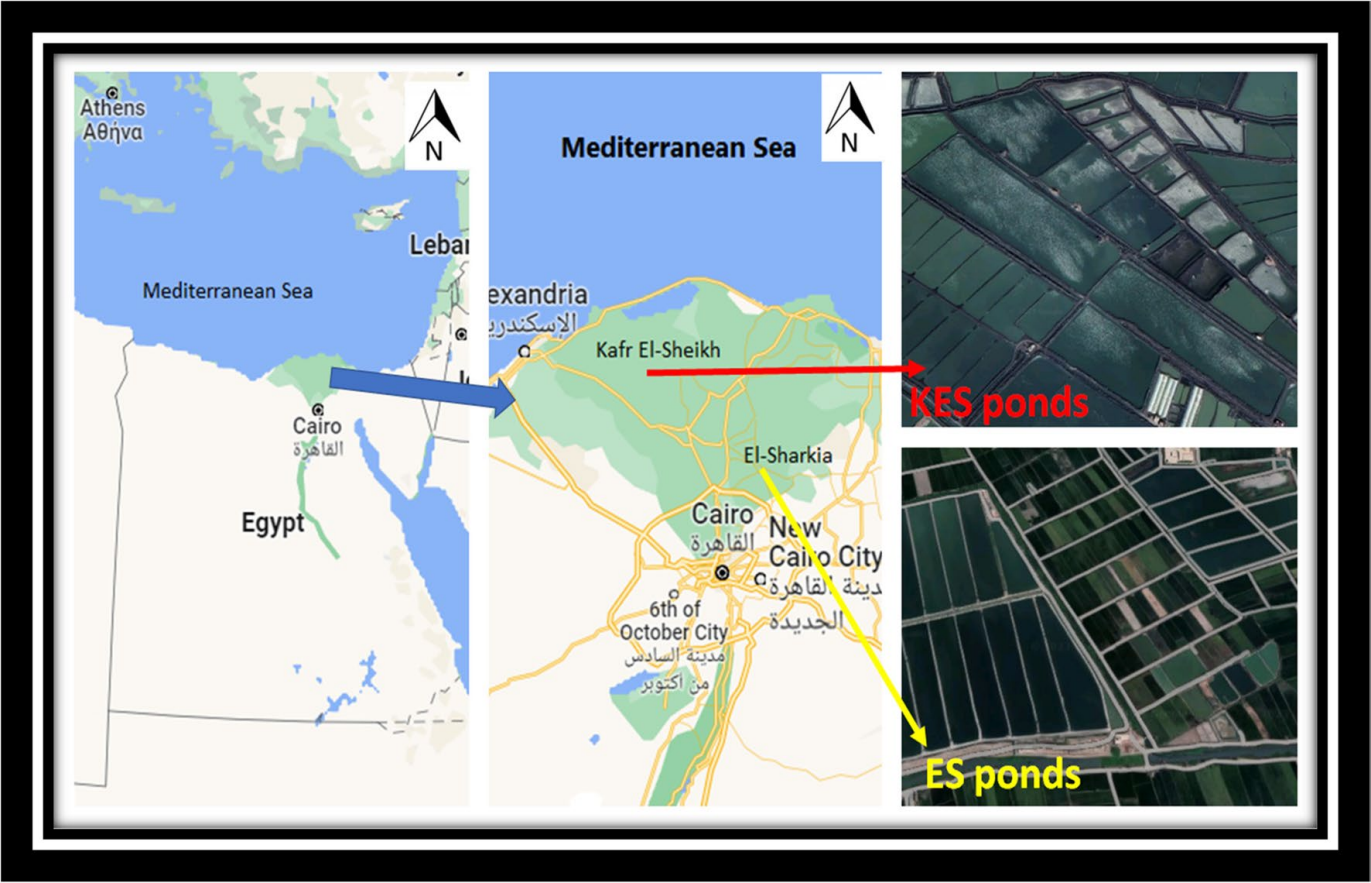
Maps images showing the two fish farms and the location of the sampling fishponds at Kafr El-Shaikh and El-Sharkia provinces

This investigation was performed at the most productive aquaculture sites in Egypt; the first farms were in the ES governorates (*n* = 7 ES fishponds, total of 18 feddan) at latitude and longitude (30.67305450 and 31.15932470, respectively), and the second ponds were in the KES governorates (*n* = 8 KES fishponds, total of 20 feddan) at latitude and longitude (31.30854440 and 30.80394740, respectively). Bolti fish is the local name of tilapia species (*Oreochromis niloticus*) while Topara fish is the local name of mullet species (*Chelon ramada*). Fish from both ponds were cultivated in polyculture and semi-intensive ponds and received their freshwater from the agricultural drainage system (Fig. 1). Bolti fish samples have an average weight and standard length of 271.26 ± 16.67 g and 18.17 ± 5.67 cm, respectively while, Topara fish samples have average weight () and standard length (). During the harvest season, samples of water, sediment, and fish (Bolti fish, *Oreochromis niloticus*, Topara fish, *Chelon ramada* (*n* = 3/pond)) samples were collected from the selected ponds and brought to the marine biology lab for further analysis.

### HMs Levels Investigations

Water, sediment and fish tissues (spleen, muscle, liver, intestines, and gills) samples were prepared, digested and diluted in detail by Abbas et al. [2] according to AOAC [1]. However, the contents of Lead (Pb), Copper (Cu), Iron (Fe), Cadmium (Cd), Manganese (Mn), and Zinc (Zn) have been measured in the diluted solutions of sediment, water, and fish tissues, using an ICP-OES instrument (inductivelycoupledplasmaopticalemissionspectrometer, 4300 DV, Perkin-Elmer, Shelton, USA). Moreover, HMC in water is expressed in ppb (µg/L), while HMC in sediments and tissues is expressed in ppm dw-b (µg/g, dw-b) on a dry weight basis.

### Metal Pollution Index (HMC-MPI)

The contamination levels of HMC in water, sediment, and in the tissues of Botti, *O. niloticus* and Topara, *C. ramada* were assessed using the HMC-MPI [12, 32] to measure the contamination degree in sediment, water, and in the studied fish tissues. The HMC-MPI was estimated using the following equation:

$${\text{HMC}}-{\text{MPI}}={\left(\mathrm{Zn\;content}\times \mathrm{Fe\;content}\times \mathrm{Cu\;content}\times \mathrm{Pb\;content}\times \mathrm{Cd\;content}\times \mathrm{Mn\;content}\right)}^{1/6}$$

The contamination level is safe degree when the MPI-HMC value is less than 1, the MPI-HMC is between 1.0 and 2.0, conditions are categorized as slightly contaminated, 2.0 to 3.0, moderately to severely contaminated, 3.0 to 5.0, severely contaminated, and > 10 heavily contaminated.

### Haematological, Biochemical, and Immunological Investigations

Regarding the evaluation of the several blood variables, sampling was performed. Haematology samples were obtained from the caudal vein of five fish that were anesthetized with clove oil (50 μL/L) using a syringe filled with EDTA blood, an anticoagulant. However, the biochemical and immunological samples were collected without the use of anticoagulants, centrifugation (3000/15 min.) at room temperature to obtain serum, and then stored at freezing point (-20 °C) until analysis. Fish haematological parameters (packed cell volume, white blood cellsred blood cells, blood cell indices, and haemoglobin levels,) were measured in EDTA blood samples according to Brown, [15], Van Kampen and Zijlstra [71], Dacie and Lewis [16]. Fish biochemical parameters (total protein (TP) and albumin (ALB) and activities of alanine and aspartate aminotransferase) were detected in blood-serum samples according to Henry [31], Reitman and Frankel [53], globulin level (GLO) was obtained by subtracting the ALB level from the TP level. Fish immune parameters (lysozyme activity, total immunoglobulin and complement C3 activities) in blood-serum samples were measured as described by Ellis [24], Siwicki and Anderson [59], and Tang et al. [62].

### Health Risk Assessment (HRA)

We employed a technique established by the USEPA (2018) to evaluate the risk to human health of HMC consumed by ingestion of the muscle of the investigated fish. The estimated daily intake (EDI-HMC), non-carcinogenic (THQ-HMC and HI-HMC indexes) and carcinogenic indexes (CR-HMC) of HMC were all performed by detecting the HMCs in the Bolti and Topara muscle from the studied ponds (Table 1S).

## Statistical Evaluations

Statistics program; software, Version 22; SPSS was used to perform the statistical evaluations. The results were tested for the significant differences using a one-way analysis of variance and where significant variations were found at *P* < 0.05, post hoc Tukey tests were used, this enabled us to detect the significant variations at *P* < 0.05 between the HMC in fish tissues. To further investigate the statistical differences between the fish supplies (KES and ES fishponds) of individual HMC, the T-test (independent sample) was used. However, the Pearson correlation coefficient was calculated to investigate the relationship between all HMCs in water, sediment, and the examined fish.

## Results and Discussion

### HMCs Estimation of Water and Sediment

HMC in the water and sediments of KES and ES fishponds are represented in Table 1. Concerning the metal levels, the sequencing of results reported at *P* < 0.05 as Fe (320.67 ± 3.66 ppb) > Zn(68.49 ± 1.23 ppb) > Cu(37.02 ± 1.02 ppb) > Mn (24.99 ± 1.22 ppb) > Pb (10.72 ± 0.89 ppb) > Cd (2.94 ± 0.35 ppb) in KES fishponds water, while it was Fe (416.00 ± 7.65 ppb) > Zn (110.05 ± 1.87 ppb) > Mn (49.79 ± 0.97 ppb) > Cu (39.44 ± 0.98 ppb) > Pb (15.40 ± 0.79 ppb) > Cd (5.10 ± 0.52 ppb) in ES fishponds water. However, in the sediment, HMCs showed significant differences (*P* < 0.05) as Fe (21312 ± 654–60332 ± 214 ppm dw-b) > Mn (846.33 ± 55.51–1058 ± 35.65 ppm dw-b) > Zn (58.33 ± 1.68–160.00 ± 1.19 ppm dw-b) > Cu (18.49 ± 5.32–53.72 ± 2.66 ppm dw-b) > Pb (10.56 ± 1.36–18.06 ± 1.32 ppm dw-b) > Cd (2.31 ± 0.17–2.76 ± 0.41 ppm dw-b) in both sites. These findings were supported by El-Batrawy et al. [22] who reported that the order of the metals exhibited the highest levels for Fe and the lowest was Cd in Burullus Lake water and in water farms [50]. Concerning the pond site, the HMCs in water and sediment samples were significantly higher in ES fishponds compared to KES fishponds. In comparison with the permissible limit [75], the studied essential HMCs in the water of ES and KES fishponds were lower than the permissible limit. However, non-essential HMC, Lead levels in ES and KES fishponds water and Cd in ES fishponds water were higher than the recommended limit. On the other hand, the studied essential HMC in the sediment of ES and KES fishponds was higher than in the upper continental crust, except for Iron in ES sediment. However, non-essential HMC, Cd levels in ES and KES fishponds sediment and Pb in KES fishponds sediment were higher than the upper continental crust [56].

**Table 1:** The HMCs (Mean ± SD) in water and sediment of ES and KES fishponds

	Water samples (ppb)			Sediment samples (ppm, dw-b)		
	KES-fishponds	ES-fishponds	WHO limit	KES-fishponds	ES-fishponds	Upper continental crust
Zn	68.49 ± 1.23^b^	110.05 ± 1.87^a^	3000	58.33 ± 1.68^b^	160.00 ± 1.19^a^	52
Fe	320.67 ± 3.66^b^	416.00 ± 7.65^a^	1000	60332 ± 214^b^	21312 ± 654^a^	30980
Cu	37.02 ± 1.02 ^b^	39.44 ± 0.98^a^	1000	18.49 ± 5.32^b^	53.72 ± 2.66^a^	14.3
Pb	10.72 ± 0.89 ^b^	15.40 ± 0.79^a^	10	10.56 ± 1.36^b^	18.06 ± 1.32^a^	17
Cd	2.94 ± 0.35 ^b^	5.10 ± 0.52^a^	3	2.31 ± 0.17^b^	2.76 ± 0.41^a^	0.102
Mn	24.99 ± 1.22 ^b^	49.79 ± 0.97^a^	100	846.33 ± 55.51^b^	1058 ± 35.65^a^	527

T-test results indicated a significant level (*p* < 0.05) for the different letters between the different ponds in the water and sediment samples

### HMCs Estimation of Cultured Fish

Metal levels are important markers of the health of fish and their surrounding environment [47]. Table 2 shows the distribution of HMC in the tissues of Bolti and Topara fish from the ES and KES fishponds. Concerning the cultured species, Bolti fish tissues in the examined ponds had significantly higher Fe, Cu, Pb, and Cd contents than Topara tissues. However, the Zn content in Bolti tissues was significantly higher than in Topara tissues, apart from muscle, which was significantly lower in Bolti tissues than Topara tissues. Additionally, the Mn content in Bolti tissues was significantly higher than that of Topara tissues, except for KES gills. However, the HMC of Bolti fish was higher than that of Topara fish in each of the studied ponds. HMC levels in different fish species vary according to their environmental requirements, physiological activity, and feeding habits [74].

**Table 2:** Heavy metal concentrations (HMC; Mean ± SD, ppm dw-b) in the tissues of Bolti and Topara fish from ES and KES fishponds

	Bolti tissues					Topara tissues				
	Liver	Gills	Spleen	Intestine	Muscles	Liver	Gills	Spleen	Intestine	Muscles
KES-fishponds										
Zn	43 ± 1.23^bB^	45 ± 1.21 ^aB^	31 ± 0.98 ^cB^	25.9 ± 0.89 ^dB^	15 ± 0.86 ^fB^	33 ± 1.42^bB^	38 ± 1.09 ^aB^	27 ± 1.02 ^cB^	23.8 ± 1.02 ^dB^	20 ± 1.02 ^fB^
Fe	100.5 ± 2.33 ^bB^	111 ± 2.32 ^aB^	70 ± 1.83 ^cB^	56 ± 1.32 ^dB^	39 ± 1.21 ^fB^	53.65 ± 1.65 ^bB^	70.3 ± 2.18 ^aB^	49.66 ± 1.88 ^cB^	38.65 ± 1.54 ^dB^	25.36 ± 1.14 ^fB^
Cu	29.35 ± 1.15 ^bB^	32.3 ± 0.98 ^aB^	23.65 ± 1.22^cB^	25.65 ± 0.96 ^dB^	19.32 ± 0.89 ^fB^	25.63 ± 0.15 ^bB^	31.2 ± 1.36 ^aB^	18 ± 1.18 ^dB^	23.65 ± 1.06 ^cB^	15 ± 0.95 ^fB^
Pb	2.55 ± 0.12 ^aB^	1.23 ± 0.02^cB^	2.05 ± 0.21^bB^	1.07 ± 0.18 ^dB^	0.66 ± 0.03 ^fB^	0.97 ± 0.14 ^cB^	1.04 ± 0.41 ^aB^	1.0 ± 0.07 ^bB^	0.15 ± 0.01 ^dB^	–
Cd	0.66 ± 0.02 ^a^	0.54 ± 0.01 ^cB^	0.57 ± 0.03^bB^	0.29 ± 0.02 ^dB^	0.08 ± 0.001^fB^	0.27 ± 0.01 ^cB^	0.39 ± 0.01 ^aB^	0.30 ± 0.04 ^bB^	0.09 ± 0.001^dB^	–
Mn	0.85 ± 0.03 ^aB^	0.67 ± 0.03 ^cB^	0.61 ± 0.05^dB^	0.75 ± 0.04 ^bB^	0.57 ± 0.01 ^fB^	0.52 ± 0.01 ^cA^	0.70 ± 0.05 ^aA^	0.46 ± 0.01 ^dA^	0.60 ± 0.03^bA^	0.41 ± 0.01 ^fA^
ES-fishponds										
Zn	45 ± 1.32 ^bA^	47 ± 1.41 ^aA^	37 ± 1.05 ^cA^	30 ± 1.02 ^dA^	18 ± 1.04 ^fA^	35 ± 1.22 ^bA^	41 ± 1.12 ^aA^	33 ± 0.90 ^cA^	27 ± 1.14 ^dA^	23 ± 1.07 ^fA^
Fe	126.3 ± 3.21 ^bA^	132.3 ± 2.86 ^aA^	77 ± 1.92 ^cA^	67 ± 1.87 ^dA^	40 ± 1.07 ^fA^	63.65 ± 1.35 ^bA^	77.65 ± 2.04 ^aA^	57.26 ± 1.57 ^cA^	45.65 ± 1.87 ^dA^	31.32 ± 1.08 ^fA^
Cu	30.53 ± 1.08 ^bA^	35.21 ± 1.05 ^aA^	25.36 ± 1.09^cA^	26.35 ± 0.89 ^dA^	22.32 ± 0.54 ^fA^	29.32 ± 1.04 ^bA^	33.25 ± 1.21 ^aA^	20 ± 1.07 ^dA^	25.36 ± 1.02 ^cA^	17 ± 0.47 ^fA^
Pb	3.55 ± 0.23 ^aA^	1.66 ± 0.03 ^cA^	2.33 ± 0.31^bA^	1.55 ± 0.21 ^dA^	0.96 ± 0.02 ^fA^	1.03 ± 0.15 ^cA^	1.56 ± 0.32 ^aA^	1.1 ± 0.06 ^bA^	0.36 ± 0.02 ^dA^	0.09 ± 0.001 ^f^
Cd	0.67 ± 0.01 ^a^	0.57 ± 0.01 ^cA^	0.60 ± 0.02^bA^	0.44 ± 0.06 ^dA^	0.10 ± 0.003 ^f^	0.34 ± 0.03 ^cA^	0.44 ± 0.06 ^aA^	0.37 ± 0.02 ^bA^	0.21 ± 0.01^dB^	–
Mn	0.99 ± 0.05 ^aA^	0.75 ± 0.03 ^cA^	0.66 ± 0.04^dA^	0.85 ± 0.03 ^bA^	0.61 ± 0.02 ^fA^	0.26 ± 0.02 ^cB^	0.53 ± 0.01 ^aB^	0.20 ± 0.01 ^dB^	0.42 ± 0.02 ^bB^	0.18 ± 0.01 ^fB^

T-test results indicated a significant level (*p* < 0.05) for both the different capital letters between different ponds in the same tissues and metals. However, ANOVA revealed that the different small letters between different tissues in the same metals and ponds were statically significant (*p* < 0.05)

Concerning the pond site, Bolti and Topara fish cultivated together had significantly higher contents of Zn, Fe, Cu, and Pb levels in ES fishponds than in KES fishponds (T-test, *p* < 0.05). However, the Cd content of Bolti and Topara fish was significantly lower in KES fishponds than in ES fishponds, except for Bolti fish liver, which was non-significant. Additionally, Mn content in Bolti fish in ES fishponds was significantly higher than in KES fishponds, whereas Mn content in Topara fish in ES fishponds was significantly lower than in KES fishponds (T-test, *p* < 0.05). The water source of the studied ponds is agricultural drainage water. However, the ES province included a large number of anthropogenic activities and industrial wastes that were discharged into the canals and drains than in KES province. Thus, cultured species in ES fishponds located in ES province with higher polluted levels of HMC than those in KES fishponds. Concerning the tissue sequence in each HMC, the Bolti tissues in both ponds are arranged in the following order: gills > liver > spleen > intestines > muscle for Zn and Fe; liver > spleen > gills > intestines > muscle for Pb and Cd; gills > liver > intestines > spleen > muscle for Cu; and liver > intestines > gills > spleen > muscle for Mn. However, in Topara tissues, the arrangement of HMC was gills > liver > spleen > intestines > muscle for Cu, Zn, and Fe; gills > intestines > liver > spleen > muscle for Mn; and gills > spleen > liver > intestines for Cd in both ponds. On the other hand, lead in Topara tissues has the following sequence: gills > spleen > liver > intestines in KES fishponds, and gills > spleen > liver > intestines > muscle in ES fishponds. Furthermore, the studied essential HMC (except Mn) of Bolti and Topara fish accumulated at the highest levels in the gills and liver. However, non-essential HMC detected the maximal levels in both the liver and spleen of Bolti fish and in both the gills and spleen of Topara fish. The HMC in an organism's tissues depends on several factors, including its species, sex, age, level of exposure to pollution, and water quality [40]. Fish kidneys, liver, and gills are examples of metabolically active organs that accumulate more HMC than muscle and skin, which are examples of metabolically fewer active organs [11, 42]. Moreover, the minimal levels of studied essential and non-essential HMC were recorded in muscles, and they also stored the least amount of HMC [55]. These findings are supported by Badr et al. [14] whom revealed that the HMC in tissues of Tilapia fish recorded the maximal levels in the liver followed by fish gill and reached the minimal level in the muscle [50]. The higher level of HMC accumulation detected in fish livers provides a useful biomarker of HMC water pollution [21], as a source of metal metabolic processes [83] the natural presence of metallic-binding proteins in hepatocyte tissues includes metallothioneins. This finding was widely reported in several fish species [3, 10, 57, 60, 65, 67].

Concerning the HMCs, the HMCs in the Bolti tissues of the studied ponds were arranged as: Fe > Zn > Cu > Pb > Mn > Cd for the liver, gills, spleen, and intestines, while it was Fe > Cu > Zn > Pb > Mn > Cd for muscle. However, there was abundance in the Topara tissues of the studied ponds as Fe > Zn > Cu > Pb > Mn > Cd for the liver, gills, and spleen, while it was Fe > Zn > Cu > Mn > Pb > Cd for the intestines. Otherwise, the Topara muscle has these arrangements: Fe > Zn > Cu > Mn > Pb in ES fishponds and Fe > Zn > Cu > Mn in KES fishponds. The accumulation of Iron in tissues of the cultivated fish increased more than the accumulation of other HMCS. These may be associated with the elevation of total dissolved Iron in the fishpond water and lakes, which could improve the free Iron content and hence HMC absorption by various organs. These observations are in agreement with Tayel et al. [63] and Al-Halani et al. [10] and Radwan et al. [50]**.** Furthermore, Cu, Fe, and Zn in Bolti and Topara fish exhibited higher essential metal content in most cases; this can be explained by the fact that these metals are essential for many biological processes and are consequently included in fish feed [58], suggesting that cultivated fish have higher levels of essential HMC [25, 81]. The maximal levels of toxic HMCs (Pb and Cd) in Bolti and Topara fish had, suggesting this may be due to their shortened lifespan and lower exposure levels (within 180 days) [45]. Furthermore, Fallah et al. [25], Kim et al. [36] Yildiz [79], and Simukoko et al. [58] revealed that diets with animal protein sources had much higher concentrations of HMC, especially those that are essential for human health such as Zinc, Iron, and Copper. The essential HMCs such as Zinc, Iron, Copper, and Manganese are expressed at higher levels due to their biological roles in comparison with the toxic HMCs, Cadmium and Lead, which are biologically insignificant and present at much lower levels in fish tissues. Numerous scholars brought up comparable circumstances [10, 20, 64, 66, 68].

In general, HMCs in the tissues of Bolti fish cultivated in KES fishponds exhibited the maximal level in the gills for Iron metal (111 ± 2.32 ppm dw-b) and the minimal level recorded in muscle for Cd metal (0.08 ± 0.001 ppm dw-b) while HMCs in the tissues of Topara fish fluctuated between 70.3 ± 2.18 ppm dw-b in the gills for Iron and 0.09 ± 0.001 ppm dw-b in the intestine for Cd metal. However, in KES fishponds, HMCs in the tissues of Bolti fish revealed the maximal value in the gills for Iron metal (132.3 ± 2.86 ppm dw-b) and the minimal value recorded in muscle for Cd metal (0.10 ± 0.003 ppm dw-b) while HMCs in the tissues of Topara fish varied from 77.65 ± 2.04 ppm dw-b in the gills for Iron to 0.18 ± 0.01 ppm dw-b in the muscle for Mn metal. According to the permissible limit [76], Zn, concentrations in tissues of two fish cultivated in both studied ponds were lower than the permissible limit of 40 ppm WHO-FAO [76] except in the liver, gills of Bolti fish of both studied ponds and gills of Topara fish cultured in ES fishponds which were higher than the permissible limit. However, Iron levels were decline than the permissible limit (100 ppm) in all tissues of the two cultivated fish except the liver and gills of Bolti fish which were higher than the permissible limit WHO-FAO [76]. Moreover, Cu, concentrations in the muscle, liver, intestines, and spleen of Bolti and Topara fish inhabiting ES and KES fishponds were lower than the permissible limit of 30 ppm while it was higher in gills WHO-FAO [76]. Except for the liver and spleen of Bolti fish, Lead concentrations in the two cultivated fish tissues were lower than the permissible limit of 2 ppm according to the World Health Organization WHO-FAO [76]. Furthermore, Cd and Mn, concentrations in all tissues of Bolti and Topara fish inhabiting ES and KES fishponds declined than the allowed limit (1 ppm) according to the World Health Organization WHO-FAO [76].

### Pearson Correlation Coefficients

Table 3 displays the findings of a hierarchical cluster analysis of the water, sediment, and fishponds using six HMC Pearson correlation coefficients**.** High-positive relationships between HMCs are considered to have shared origins, whereas extremely negative relationships between HMCs are considered to have different origins [8]. However, correlations between HMCs can reveal information about their origin and their association. A significant positive correlation was observed between the following metals, implying that they may have originated from similar sources: Cu, Fe, Pb, and Zn for KES fishponds water; Cu, Fe, Mn, Cd, and Pb for ES fishponds water; Cu, Fe, Mn, and Pb for the sediment of the studied ponds; Cd for KES fishponds Bolti samples; Cu and Mn for ES fishponds Bolti; Cd and Pb for KES fishponds Bolti; Cu and Mn for ES fishponds Bolti; Cd and Pb for KES fishponds Topara samples; and Mn for ES fishponds Topara samples. The relationships between the different HMCs may stem from the similarities in the cumulative behaviour of the different HMCs in fish and their interactions [54]. According to Kumar et al. [37]**,** the significant relationships between metals may be due to a shared origin for their occurrence and suggest similar biogeochemical processes for subsequent accumulation in fish muscle. In addition, the six HMCs of the water, sediment, and tissues of fishponds were separated into two clusters: Cu, Fe, Pb, and Zn; Cd and Mn for water and Bolti samples; Cu, Zn, and Cd; Fe, Mn, and Pb for sediment samples; and Cu, Fe, and Zn; Mn, Cd, and Pb in the case of Topara samples. However, in ES fishponds samples, it was also divided into two clusters: Cu, Fe, Mn, and Zn; Cd and Pb for Topara and Bolti samples; Zn; Cu, Fe, Mn, Cd, and Pb for water; and Zn-Cd; Cu, Fe, Mn, and Pb for sediment samples from ES fishponds.

**Table 3:**
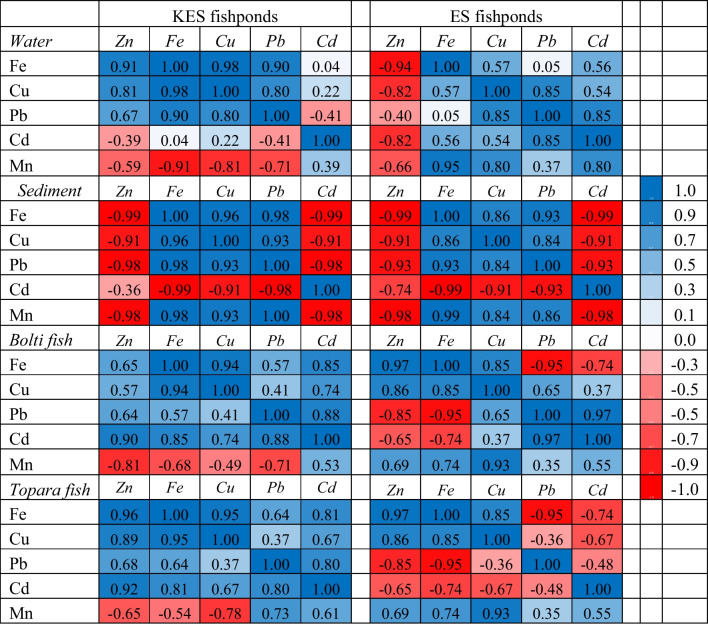
Pearson correlation coefficients based on HML in the water, sediment, Bolti, and Topara from different fishponds

### Bio-Accumulation and Bio-Sedimentation Factors

**Fig. 2:**
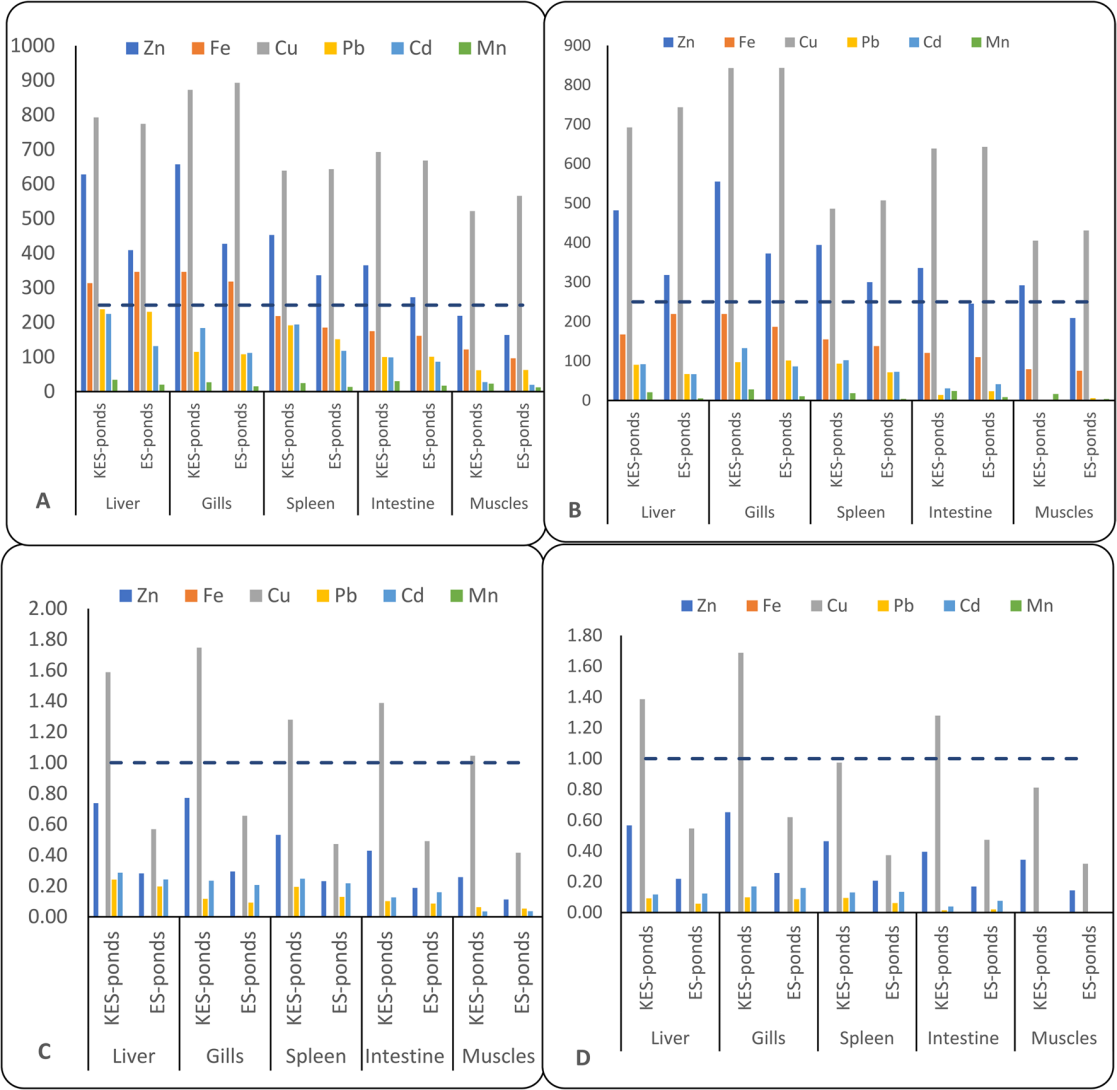
Bio-sedimentation and bio-accumulation factors of HMC in the tissues of Bolti and Topara fish from ES and KES fishponds: A-BAF-HMC Bolti,: B-BAF-HMC Topara, C-BSF-HMC Bolti,: D-BSF-HMC Topara

Bio-accumulation (BAF-HMC) and bio-sedimentation factors (BSF-HMC) of HMC in the Topara and Bolti fish inhabiting KES and ES fishponds are shown in Fig. 2. The positive BAF-HMC values reported that the cultivated fish could derive HMCs from water [3]. The highest BAF-HMC values were observed for BAF-Cu, which ranged from 405 to 893, whereas the lowest BAF-Mn values were observed, with BAF-HMC values ranging from 3.6 to 34. The maximum values of BAF-HMC were recorded in Bolti tissues, and the lowest were observed in Topara tissues. However, it was higher in the tissues of fish inhabiting ES fishponds than in KES fishponds. The BAF-HMC values in Topara tissues exhibited their maximum level in the gills, and the minimum was observed in the muscle. However, in the Bolti fish, the highest BAF-HMC values were recorded in the gills for BAF-Zn, BAF-Fe, and BAF-Cu and in the liver for BAF-Pb, BAF-Cd, and BAF-Mn, while the lowest values were observed in the muscle. The diversity of biochemical and physiological characteristics of organs as well as the level of environmental contamination affect BAF-HMC values [70]. The order of fishpond water BAF-HMC was BAF-Fe > BAF- Zn > BAF-Cu > BAF-Mn > BAF-Pb > BAF-Cd, while the order of BAF-HMC was BAF-Cu > BAF-Zn > BAF-Fe > BAF-Cd > BAF-Pb > BAF-Mn in the Topara fish and BAF-Cu > BAF-Zn > BAF-Fe > BAF-Pb > BAF-Cd > BAF- Mn in the Bolti fish. This suggests that a variety of parameters, including environmental factors, the level and chemical structure of HMC, pollution degree, farm environments, age, species, feeding patterns, and metabolic rate, affects the bioavailability of HMC by fish [5, 19, 43].

Regarding the BAF-HMC values for Bolti samples, BAF-Cu in all tissues is of moderate concern (250 > BAF-HMC 1000), and BAF-Cd, BAF-Manganese, and BAF-Lead are of low concern (BAF-HMC 250) for all studied tissues. Except for muscle, where BAF-Zn was of medium concern (250 > BAF-HMC 1000), BAF-Zn is present in all tissues. While spleen, muscle, and liver Fe were of low concern (BAF-HMC 250), gills and liver BAF-Fe were of moderate concern (250 > BAF-HMC < 1000). However, in Topara fish, BAF-Cd, BAF-Mn, BAF-Fe, and BAF-Pb are of low concern (BAF-HMC < 250 > BAF-HMC < 1000 > BAF-HMC < 1000) except in muscle. The BAF-HMC provides details on the accumulation of specific metals in tissues of fish. The overall findings revealed that for almost every HMCs, metals accumulation in muscle, liver, gills, intestines, and spleen decreased in Topara fish compared to Bolti fish when comparing the BAF-HMC values of the two species. BAF-HMC changes from the environment to fish tissue depending on chemical category, organ metabolite properties, and degree of environmental contamination [13, 46, 82].

The BSF-HMC, on the other hand, measures the proportion of HMCs in sediment to those in tissues [4]. According to their BSF-HMC values, tissues are categorised into three groups: macro-concentrators (BSF-HMC values > 2), micro-concentrators (BSF-HMC values 1 to 2), and de-concentrators of metals (BSF-HMC values 1) [84]. Based on the above, the Bolti and Topara tissues from ES and KES fishponds showed that BSF-HMC of muscle, liver, gills, intestines, and spleen are de-concentrators (BSF-HMC < 1) for all metals and release the metals in sediment except Cu, tissues are micro-concentrator (1 < BSF-HMC < 2). This means that there is almost no BSF-HMC in studied fish tissues. Additionally, Bolti tissue in the study ponds had the highest BSF-HMC value, whereas Topara tissue had the lowest value. However, it was higher in the tissues of fish inhabiting ES fishponds than in KES fishponds. Topara gills had the highest BSF-HMC value of any tissue, while the muscle had the lowest value. In contrast, in the Bolti fish, the liver showed the highest BSF-HMC values for Pb, Cd, and Mn, and the gills showed the lowest values for Zn, Fe, and Cu. In general, the BAF-HMC value appears to be higher than the BSF-HMC value.

### Metal Pollution Index (MPI-HMC)

**Fig. 3:**
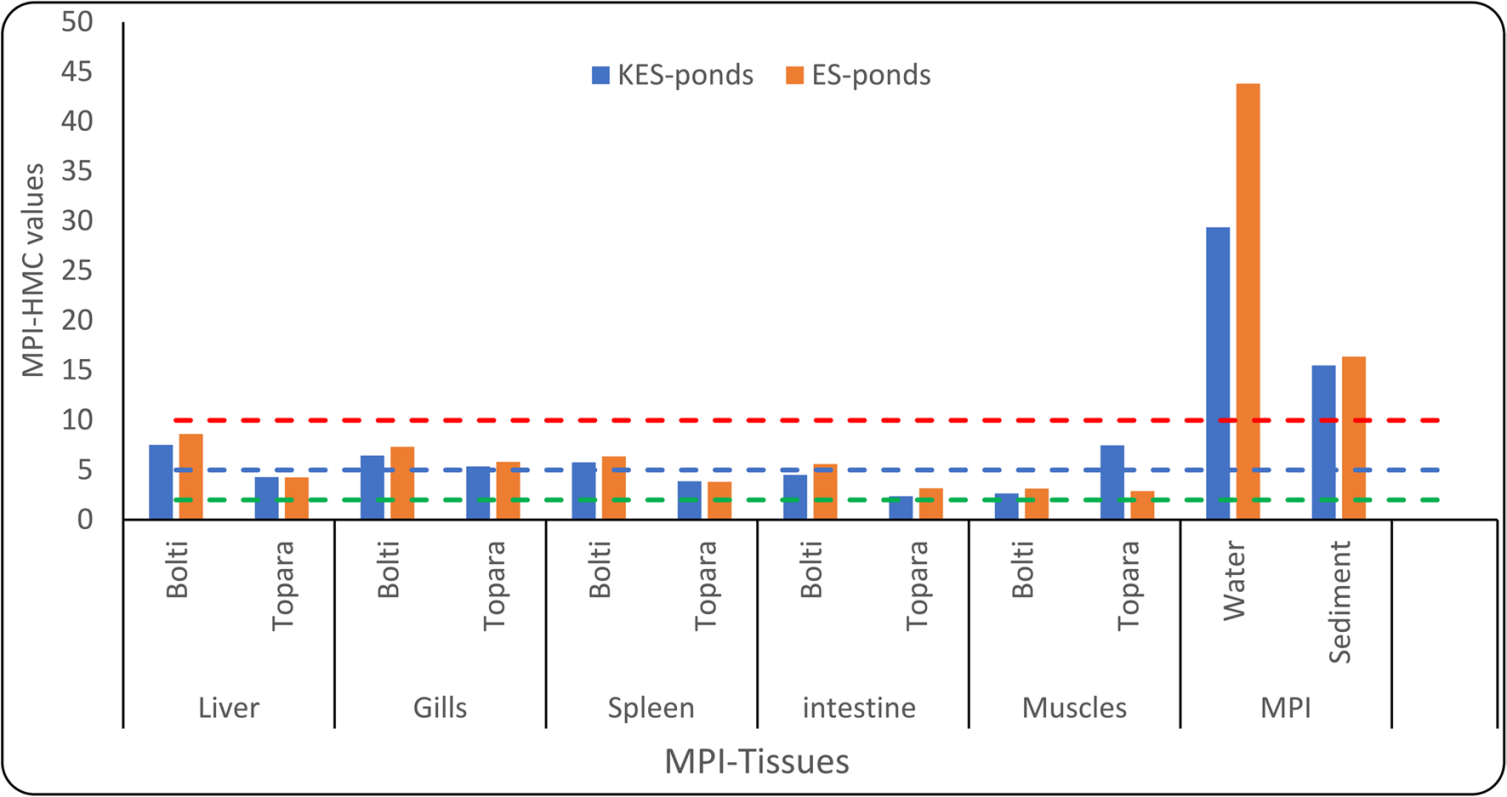
MPI-HMC in the water, sediment, and tissues of Bolti and Topara fish from ES and KES fishponds

The high MPI-HMC score indicates that the fish species has significant cumulative HMC accumulations [49] and poses a potential risk to public health is fish consumption with a high MPI-HMC value [5]. Figure 3 displays the presence of MPI-HMC in the samples of water, sediment, and tissues (Bolti and Topara fish) from the ES and KES fishponds. The MPI-HMC values of water, and sediment were over 10, this suggested the water and sediment of the different fishponds were heavily contaminated with heavy metal levels and it was higher in ES fishponds than in KES fishponds. Except for Topara muscle, MPI-HMC samples measured higher levels of metal pollution for all samples from ES fishponds compared to the KES fishponds. Therefore, this result shows that ES fishponds are more polluted by the investigated metals than KES fishponds.

### Haematological, Immunological, and Biochemical Alterations

Blood variables are widely employed to examine fish well-being due to the fact they are easily determined and strongly related to environmental and physiological fish behavior [17]. Haematological indices are the most critical parameters used as indicators of the health of fish [48]. Haematological, immunological, and biochemical examination of Bolti and Topara fish are mentioned in Table 4. The heamatology of Bolti and Topara cultured in ES fishponds detected significantly decrease (*p* < 0.05) in Hb, MCHC, RBC count, PCV, and MCH compared with those fish cultured in KES fishponds. Conversely, WBC count and MCV showed significantly increase (*P* < 0.05) in fish cultured in ES fishponds in comparison to these fish cultured in KES fishponds. Insufficient levels of the studied haematological markers could indicate hemodilution or anaemia attributed to metabolism failure [72]. In addition, serum biochemical indicators demonstrated a significant increase (*P* < 0.05) in ALAT and ASAT activity and a significant decrease in total protein, albumin, and globulin in cultivated fish in ES fishponds compared to these fish cultured in KES fishponds. The decrease in protein levels has been linked to physiological deficits, malnutrition, infections, and haemodilution, which decreased absorption of nutrients [18]. Also, protein deficiency in fish can be caused by changes in water quality due to waste from many causes, such as contamination in industrial and agricultural water supplies [77]. However, immunological assay declared a significant decrease (*P* < 0.05) in total Ig (mg/mL), complement C3 (mg/mL), and lysozyme (U/mL) in Bolti and Topara samples cultured in ES fishponds compared to fish cultured in KES fishponds. These results correspond to the HMC and their contamination degree and confirm that the HMC contamination levels affected the physiological and immune status of Bolti and Topara fish cultured in ES fishponds compared to cultured fish in KES fishponds.

**Table 4:** Hematological, immunological, and biochemical alterations (Mean ± SE) of Bolti and Topara from ES and KES fishponds

		Bolti sites		Tobara sites
	KES-fishponds	ES-fishponds	KES-fishponds	ES-fishponds
Hematological parameters				
RBCs (× 10^6^ cell / mm^3^)	2.94 ± 0.13^a^	2.09 ± 0.18^b^	3.12 ± 1.02^a^	2.28 ± 0.71^b^
WBCs (× 10^3^ cell / mm^3^)	14.11 ± 0.74^b^	20.83 ± 0.51^a^	15.77 ± 1.36^b^	22.14 ± 2.81^a^
Hb (g/dl)	10.61 ± 0.42^a^	6.06 ± 0.28^b^	12.14 ± 2.04^a^	7.67 ± 1.27^b^
PCV (%)	31.53 ± 1.14^a^	23.69 ± 1.42^b^	37.28 ± 1.93^a^	28.45 ± 2.35^b^
MCV (fL)	6.39^b^ ± 107.24	4.48^a^ ± 113.35	119.49 ± 3.78^b^	124.78 ± 4.28^a^
MCH (pg)	2.04^b^ ± 36.09	1.04^a^ ± 29.00	38.91 ± 1.04^b^	33.64 ± 1.69^a^
MCHC (%)	33.65 ± 1.28^b^	25.58 ± 0.89^a^	32.56 ± 2.49^b^	26.96 ± 2.07^a^
Biochemical parameters				
Total protein (g/dl)	3.14 ± 0.3^a^	2.66 ± 0.49^b^	4.40 ± 0.237^a^	3.40 ± 0.237^b^
Albumin (g/dl)	1.73 ± 0.54^a^	1.43 ± 0.33^b^	2.90 ± 0.033^a^	2.27 ± 0.033^b^
Globulin (g/dl)	1.41 ± 0.26^a^	1.12 ± 0.50^b^	1.50 ± 0.25^a^	1.13 ± 0.25^b^
AST (U/L)	39.2 ± 0.77^b^	44.2 ± 0.64^a^	30.2 ± 0.74^b^	43.82 ± 0.89^a^
ALT (U/L)	20.08 ± 0.54^b^	22.5 ± 0.58^a^	19.75 ± 0.39^b^	21.5 ± 0.66^a^
Immunological parameters				
Total Ig (mg/mL)	11.87 ± 0.23^a^	7.87 ± 0.23^b^	17.87 ± 0.23^a^	10.87 ± 0.23^b^
Complement C3 (mg/mL)	33.33 ± 0.75^a^	25.33 ± 0.15^b^	40.25 ± 0.66^a^	30.33 ± 0.65^b^
Lysozyme (U/mL)	45.65 ± 2.06^a^	39.08 ± 1.55^b^	43.90 ± 1.95^a^	35.26 ± 1.15^b^

T-test results indicated a significant level (*p* < 0.05) for the different letters between the different ponds in the same species

### Human Health Hazard

**Fig. 4:**
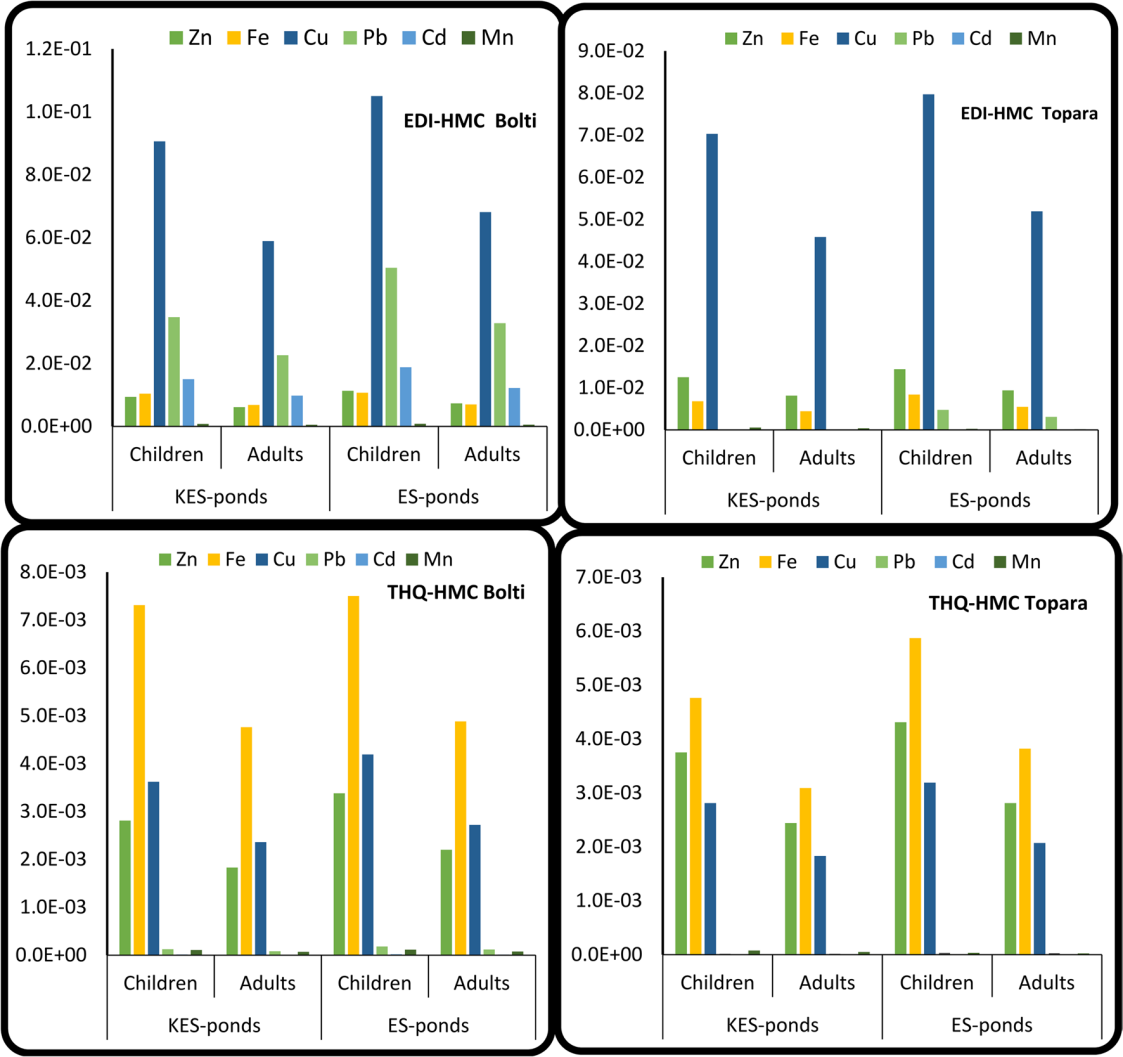
Values of EDI-HMC (mg/kg/day), THQ-HMC and HI of HMC in the muscles of Bolti and Topara fish from ES and KES fishponds

Estimated daily exposure to HMC by consumption of fish high in HMC was required to avoid any adverse impacts on human health over the duration of a person's lifetime [51]. The allowable quantities of HMC were established using the EDI-HMC [35]. EDI-HMC values (mg kg-1 day-1) in the Bolti and Topara muscles cultured in ES and KES fishponds are shown in Fig. 4. Regarding species, the EDI-HMC value in the Bolti muscle cultured in the studied ponds was higher than that in the Topara muscle, which implied that the Bolti fish establish a high rate of exposure for customers (children and adults) through HMC consumption. The EDI-Fe, EDI-Zn, EDI-Cu, EDI-Ni, EDI-Cd, and EDI-Pb values in the Bolti and Topara muscles from the different fishponds were less than the permissible tolerable daily intake, PTDI, based on limits according to FAO-WHO guidelines. The PTDI values of Fe, Zn, Cu, Mn, Cd, and Pb are 50, 70, 50, 4E-02, 3E-03, and 3E-02 mg/kg/day, respectively [27, 33]. The EDI-HMC value was higher in fish cultured in ES fishponds in comparison to samples cultured in KES fishponds, which implied that the fish of ES fishponds exert the highest exposure in both children and adults through the HMC intake.

**Fig. 5:**
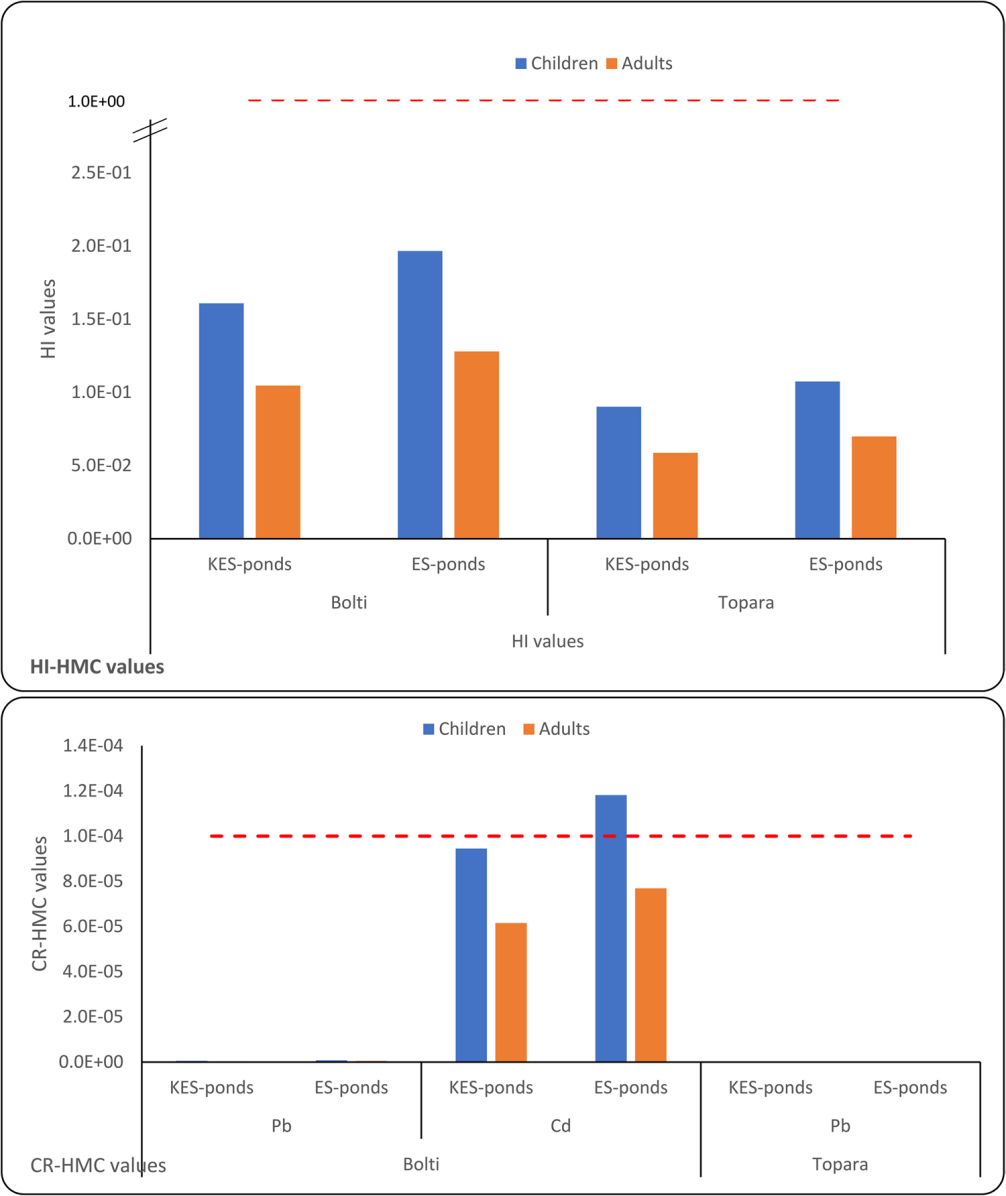
HI-HMC values and CR-HMC values of Bolti and Topara muscles from ES and KES fishponds

The THQ-HMC allowable threshold limit is 1.00. The degree of exposure was still lower than the advised dosage when THQ-HMC was below the unit limit; hence, exposure to HMC won't have a negative impact on lifetime intake [78]. However, the target hazard quotient (THQ-HMC) values for Mn, Iron, Cd, Copper, Lead and Zinc in the muscles of the studied species cultured in KES and ES fishponds are illustrated in Fig. 4. The THQ-HMC values in the muscles of the studied species were all below 1. Additionally, under USEPA [69], the THQ-HMC estimate for each HMC is added up and represented as a hazard index, HI-HMC, to measure the total possible non-carcinogenic effects caused by many metals. The THQ-HMC data were used to calculate the risk index (HI-HMC) for both adults and children consuming the two species; if the HI-HML value was > 1, the effect on consumers had an adverse effect, as suggested by Lei et al. [38]. The HI-HMC values in the Bolti and Topara muscles of the two ponds were both less than one, indicating no non-carcinogenic risk for human intake occurred (Fig. 5).

The non-essential HMC Pb and Cd may increase the risk of human cancer (CR-HMC, [39]). Furthermore, the carcinogenic index (CR-HMC) values of Cadmium and Lead in the Bolti and Topara muscles of the two ponds were determined for eaters (adults and children), and the results are presented in Figs. 5. The CR-Pb values in Bolti and Topara muscles were less than 1E − 6 for both children and adult consumers, suggesting that the CR-Pb was safe [73]. However, Cd poses a cancer risk (CR-Cd) to children’s consumers of Bolti and Topara muscle in ES fishponds, as the CR-Cd values were higher than the acceptable limit value of 1E − 6 [35]. Generally, the non-cancer (THQ-HMC and HI-HMC) and cancer (CR-Pb and CR-Cd) risks of HMCs in muscles of cultivated Bolti and Topara fish were elevated in ES fishponds than in KES fishponds. This suggests that fish cultivated in ES fishponds are more polluted with HMCs than fish cultivated in KES fishponds. Regarding to previous literatures, children’s consumers were more prone to CR-HMC exposure, Ahmed et al. [7], Abtahi et al. [6], Noman et al. [44] and Raknuzzaman et al. [52]

## Conclusion

The Zinc, Fe, Cu, Pb, Cd, and Mn concentrations were presented in varying levels in tissues, sediment and water samples, with the accumulation rate depending on the species (Bolti and Topara), the tissues (spleen, muscle, liver, intestines, and gills), and fishponds (ES and KES fishponds). The findings mentioned that it was a significantly difference (T-test, *p* < 0.05) between the studied fishponds on HMC, recorded in tissues, water, and sediment, with elevated concentration found in ES-fishponds than in KES-fishponds. It was revealed that in Topara and Bolti fish, the HMC contents in their muscles were lower than the acceptance limits. El-Sharkia ponds had higher levels of HMC distribution and accumulation in the sediment, water, and fish tissues than KES fishponds, indicating that ES fishponds have higher levels of pollutants in comparison to KES fishponds. This may be reflected in the differences between Kafr El-Sheikh and Sharkia fishponds in terms of the haematological, immunological, and biochemical changes that occur there, as well as in the results on the degree of HMC contamination in both fishponds. Children and adults were both exposed to HMC through the consumption of cultivated fish in different fishponds, but no notable adverse non-carcinogenic risks to the consumers were identified, as the estimated values of THQ-HMC and HI-HMC were < 1. Nonetheless, the CR-Pb values for both children and adult consumers were less than the allowed limit, meaning that the carcinogenic hazard caused by Pb was safe, while Cd poses a cancer risk (CR-Cd) to children’s consumers of Bolti and Topara muscle in ES fishponds, as the CR-Cd values were higher than the acceptable limit. These investigated areas (ES and KES) are crucial to the Egyptian aquaculture industries of Bolti and Topara. Future heavy metal contamination in fish farms should be minimised through regular monitoring and awareness-raising, thereby reducing the risk to public health. Therefore, to protect the Bolti and Topara aquaculture sectors in Egypt, more detailed studies are required with a focus on metals in fish feed, water quality, waste management, and handling processes.

### Supplementary Information

Below is the link to the electronic supplementary material.

Supplementary file1 (DOCX 15.9 KB)

## Data Availability

The data sets in this study are available from the corresponding author on reasonable request.
