# Supplementary material for: Heavy Metals Assessment and Health Risk to Consumers of Two Commercial Fish Species from Polyculture Fishponds in El-Sharkia and Kafr El-Sheikh, Egypt: Physiological and Biochemical Study
Source: Biol Trace Elem Res. 2023 Dec 22;202(10):4735–50. doi: 10.1007/s12011-023-04007-1 (PMC11338967; doi:10.1007/s12011-023-04007-1)
Supplement: Supplementary file 1 — Supplementary file1 (DOCX 15.9 KB) [file 12011_2023_4007_MOESM1_ESM.docx]

Heavy Metals Assessment and Health Risk to Consumers of Two Commercial Fish Species from Polyculture Fishponds in El-Sharkia and Kafr El-Sheikh, Egypt: Physiological and biochemical study

**Mahmoud Mahrous M. Abbas^*1^**

**Salah M. EL-Sharkawy^2^**

**Hassan R. Mohamed^3^**

**Bassem E. Elaraby^2^**

**Walaa M. Shaban^1^**

**Metwally G. Metwally^2^**

**Diaa M. G. Farrag^1^**

**^1^**Marine Biology Branch, Zoology Depart., Science Facu., Al-Azhar University, Cairo, Egypt.

^2^Zoology Depart., Science Facu., Al-Azhar University, Cairo, Egypt.

^3^Fayoum, Egypt Marine Products Processing Technology Depart., Aquaculture and Marine Fisheries Facu., Arish University, Egypt.

**--------------------------------**

*Corresponding author: **E-Mail:** [Mahmoud_Mahrous42@azhar.edu.eg](mailto:Mahmoud_Mahrous42@azhar.edu.eg%20)

Phone: 00201064303459 **ORCID**: <https://orcid.org/0000-0002-2061-4101>.

Table 1S. Estimated Daily Intake (HMC-EDI, mg kg-1 day-1), Target hazard quotient (HMC-THQ), Hazard index (HMC-HI) and Carcinogenic index (HMC-CR)

| HRA Index | Equation | Details |
| --- | --- | --- |
| HMC-EDI  (Mwakalapa et al., 2019) | HMC-EDI =(EP×IR×HMC×ER/BW×AT)×10^-3^ | where the IR is ingestion rate (IR= 41 g/day for adults and 27 g/day for children). The EP is the exposure period (70 years old).  The ER is the exposure rate (ER=365 days/year); BW is the weight of the body (70 kg for adults and 30 kg for children); and AT is an average lifespan (365 days/year x 70 years). The HMC stands for the HMCs in muscle (ppm wet wt.); the weight of fish in wet was converted to the weight of fish in dry using a coefficient of conversion (4.8 as Rahman et al., 2012). |
| HMC-THQ  (USEPA, 2018) | HMC-THQ = EDI-HMC/ORD-HMC | Where ORD-HMC is oral reference doses of HMC |
| HMC-HI **(**Cui et al., 2015) | HMC-HI = ∑ THQ-HMC (6 HMC). |  |
| HMC-CR  (Varol et al., 2017) | HMC-CR = EDI-HMC x CSF. | Where CSF is carcinogenic slope factor (CSF-Cd, and CSF-Pb were 6.3, and 0.0042 mg/kg/day, respectively). |
